# Efficient inference of homologs in large eukaryotic pan-proteomes

**DOI:** 10.1186/s12859-018-2362-4

**Published:** 2018-09-26

**Authors:** Siavash Sheikhizadeh Anari, Dick de Ridder, M. Eric Schranz, Sandra Smit

**Affiliations:** 1 Bioinformatics Group, Wageningen University, Wageningen, The Netherlands; 2 Biosystematics Group, Wageningen University, Wageningen, The Netherlands

**Keywords:** Pan-genome, Protein similarity, Homologous genes, Orthology, *k*-mer

## Abstract

**Background:**

Identification of homologous genes is fundamental to comparative genomics, functional genomics and phylogenomics. Extensive public homology databases are of great value for investigating homology but need to be continually updated to incorporate new sequences. As new sequences are rapidly being generated, there is a need for efficient standalone tools to detect homologs in novel data.

**Results:**

To address this, we present a fast method for detecting homology groups across a large number of individuals and/or species. We adopted a *k*-mer based approach which considerably reduces the number of pairwise protein alignments without sacrificing sensitivity. We demonstrate accuracy, scalability, efficiency and applicability of the presented method for detecting homology in large proteomes of bacteria, fungi, plants and Metazoa.

**Conclusions:**

We clearly observed the trade-off between recall and precision in our homology inference. Favoring recall or precision strongly depends on the application. The clustering behavior of our program can be optimized for particular applications by altering a few key parameters. The program is available for public use at https://github.com/sheikhizadeh/pantools as an extension to our pan-genomic analysis tool, PanTools.

**Electronic supplementary material:**

The online version of this article (10.1186/s12859-018-2362-4) contains supplementary material, which is available to authorized users.

## Background

Detection of homologous genes (genes that share evolutionary ancestry) is fundamental to comparative genomics, functional genomics and phylogenomics. Homologs inherited from a single gene in the last common ancestor of two species are called orthologs, while those inherited from distinct duplicated genes are called paralogs [1]. Orthologs are usually under selection pressure, which conserves their sequence, structure and function; while paralogs can diverge rapidly and lose their previous functions or achieve completely or partially new functions [2].

With increasingly evolutionary distance and/or increased data-set sizes, there will be greater sets of gene and genome changes, that can complicate orthology inference [3]. Whole-genome and segmental duplications increase genomic content, local and structural mutations lead to gene losses and gains, and horizontal gene transfers mix genomic content between species. As a result, orthology detection is increasingly difficult in higher organisms and across large evolutionary distances.

In the presence of gene duplications, orthology is not always a one-to-one relationship but rather can be a one-to-many or even many-to-many relationship [4]. As a consequence, an orthology group may contain not only orthologous pairs, but also pairs of homologs duplicated after the speciation of the two species, so-called in-paralogs. In the rest of this text we therefore use the term homology group instead of orthology group to be more precise.

To date, several databases of homology groups have been established, which need to be continually updated to incorporate new genomes [5–8]. As genomic projects are generating novel data at an unprecedented scale, the analysis of new data means that researchers have to automate the process of inferring homology in their large gene sets. Consequently, in parallel to the static databases there has been a development of standalone tools for automatic detection of homologs [9–11]. Accurate homology detection tools rely on all-pairs comparison of proteins. However, calculating all-pair similarity scores quickly becomes a major computational burden as the number of proteomes increases. As the number of eukaryotic proteomes keeps expanding in the coming years, there is a need for even more efficient homology detection methods.

Here, we present an efficient graph-based approach towards homology detection. This method extends the functionality of our pan-genomic data analysis tool, PanTools [12], which integrates genomes, annotations and proteomes in a single graph database to facilitate comparative studies at the levels of structure, variation and function [13]. The motivation of this study was to detect homology groups de novo and efficiently, in large datasets of hundreds of eukaryotic genomes. The presented method scales to large proteome sets while maintaining its accuracy and can be tuned for different application scenarios.

## Methods

**Fig. 1:**
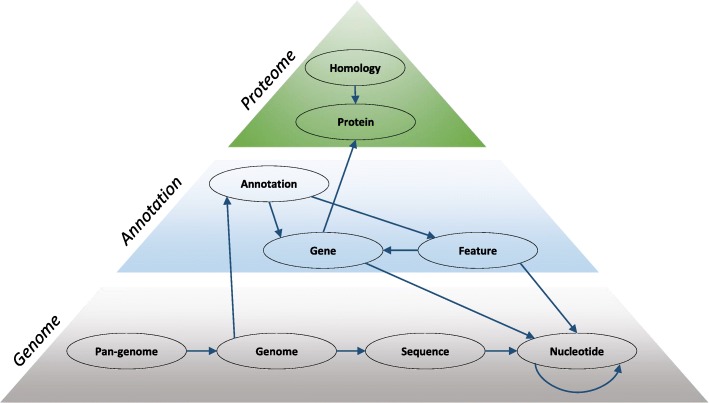
Integrating genomic data in a hierarchical pan-genome. The Neo4j graph data model allows to store different types of data in the nodes and edges of a graph

We represent a pan-genome by a hierarchy of genome, annotation and proteome layers stored in a Neo4j graph database to connect different types of data (Fig. 1). The genome layer consists of pan-genome, genome, sequence and nucleotide nodes which contain some essential information about these entities. Nucleotide nodes form the generalized de Bruijn graph [12] which enables the compression and reconstruction of the constituent genomes. The annotation layer, currently, consists of the genomic features like genes, mRNAs, etc. Finally, the proteome layer of the pan-genome is formed by proteins and homology nodes which group the homologous proteins.

Before homology detection, first the protein nodes should be stored in a pan-genome graph. Instructions for constructing a pan-genome can be found in the Additional file 1. Having the proteins available in the proteome layer of the pan-genome, we take the steps described in Algorithm 1 to cluster them in homology groups.

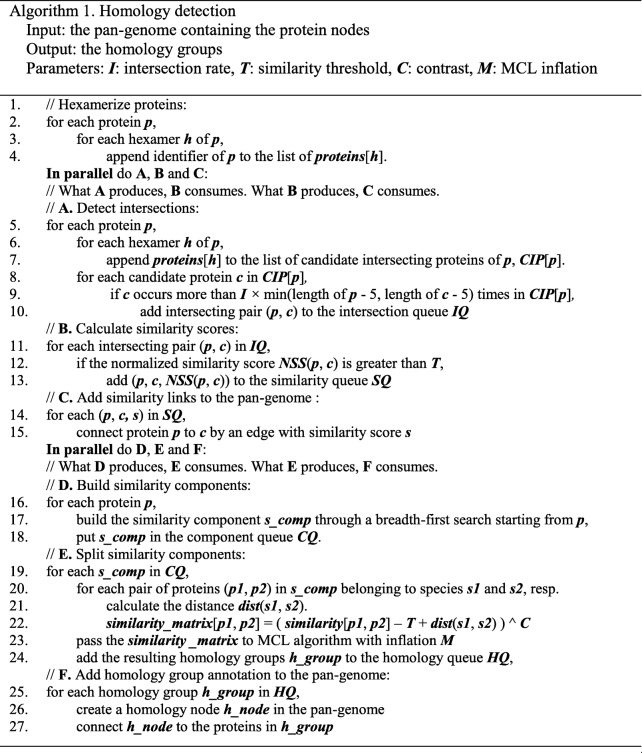



**Fig. 2:**
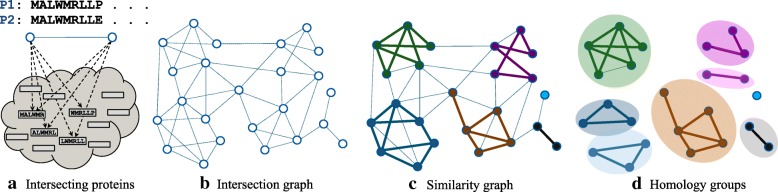
**a** An example of two intersecting proteins, P1 and P2, which share some hexamers. **b** The intersection graph is built from intersecting pairs of proteins. **c** The similarity graph consists of similarity components. Each bold edge represents a similarity score greater than the threshold (*T*). **d** Homology groups are detected in each similarity component by MCL

First, we extract the hexamers of all proteins and, for each hexamer, keep track of the proteins containing that hexamer (lines 1–4). Then, we find all pairs of intersecting proteins (lines 5–10) and calculate their similarity score by aligning them. Two proteins intersect (Fig. 2a-b) if the number of hexamers they share is greater than the product of the intersection parameter (*I*) and the total number of hexamers of the shorter protein. We connect the intersecting proteins with a similarity score greater than the similarity threshold *T* (lines 11–15) to form the similarity graph (Fig. 2c). For reasons of efficiency, we have implemented this as three parallel routines A-C, in which B consumes the output of A and C the output of B. A and C employ one working thread and B multiple threads to maximize performance. Next, all the connectivity components of the resulting similarity graph are found using a simple breadth-first search (lines 16–18). This search allows to detect not only the directly connected proteins but also those connected through a path in the graph, the potential distant homologs. Every similarity component is then passed to the MCL (Markov clustering) algorithm [14] to be possibly broken into several homology groups (lines 19–24) (Fig. 2d). MCL has been frequently employed in homology inference methods [11, 15, 16]. Finally, the members of each homology group are connected to a single homology node in the graph (lines 25–27).

### Normalizing the raw similarity scores

We compare intersecting pairs of proteins by a Smith-Waterman local alignment algorithm with an affined gap penalty (opening = − 10, extension = − 1) using the BLOSUM62 (Blocks Substitution Matrix 62) scoring matrix. After calculating the raw similarity scores, we normalize them to be independent of the protein lengths. To this end we divide each raw score by the score achieved by aligning the shorter protein to itself and multiply the result by 100; this way, the normalized similarity scores will always be less than or equal to 100. For the sake of simplicity, we use the term similarity score to refer to the normalized similarity score between pairs of proteins.

### Rescaling the similarity scores

The pairwise similarity scores of highly similar homologs, which usually lie in the same similarity component, are very close to each other. This makes it very hard for MCL to detect the underlying substructures in such similarity components. To resolve this problem, we rescale the similarity scores in three different ways (Algorithm 1, line 22). First, we subtract the value *T* from these scores to emphasize small differences for the MCL process.

Furthermore, we would like the clustering to be relatively insensitive to evolutionary sequence divergence. That is, within a similarity component pairs of homologs from two distant species should be ideally scored nearly as high as pairs from two closely related species. To achieve this, in each similarity component we calculate the average distance between each pair of species as 100 minus the average inter-species similarity score and add it to all the similarity scores between those species within the similarity component.

Finally, to increase the contrast between the final similarity scores, before the similarity component is passed to the MCL algorithm, we raise the scores to the power of *C*, the contrast parameter. This operation is similar to one round of expansion as explained in [14] and was experimentally observed to increase the specificity of the resulting clusters.

### Choice of *k*

Short peptide *k*-mers may occur in many proteins. This raises the number of intersecting proteins which will be aligned, increasing the resource consumption of the program significantly. On the other hand, long *k*-mers are more specific and decrease the sensitivity of the program in detecting the intersecting pair of proteins, thereby reducing the recall. As a result, we calculate the smallest *k* value which keeps the probability of random occurrences of a *k*-mer below a desirable probability *p*. For peptide sequences, size of the alphabet *α* = 20, and considering *L* = 30,000 the length of the largest known protein [17] and setting *p* = 0.001, the smallest suitable *k* will be 6 (see Additional file 1). Therefore, we chose to use hexamers for detecting the intersections.

To reduce the memory needs of the program and increase the specificity of the intersections, we ignore extremely abundant hexamers (for example “QQQQQQ” in the yeast datasets), which their frequency exceeds *p × n + c × m*, where *p* = 0.001, *n* is the total number of proteins, *c = 50* is an a priori estimate of the maximum number of occurrences of a hexamer in the proteome of a species, and *m* is the total number of species (proteomes). Likewise, hexamers with frequency 1 are considered rare and thereby ignored. This filtration notably improves the efficiency and the precision of the method.

### Measures of accuracy for evaluation

**Fig. 3:**
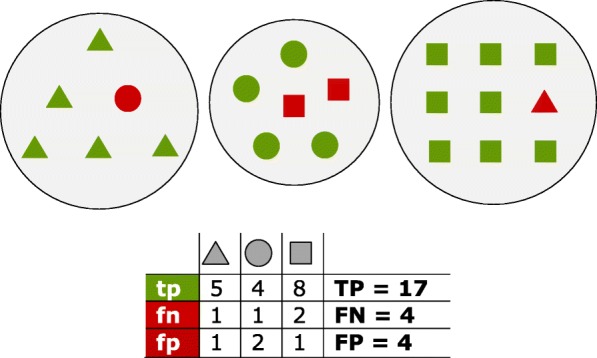
Proteins of three distinct homology groups are represented as triangles, circles and squares. Green shapes are true positives (*tp*) which have been assigned to the true group; red shapes are false positives (*fp*) for the group they have been incorrectly assigned to, and false negatives (*fn*) for their true group

To evaluate the accuracy of the method, we used the recall, precision and F-score measures as defined previously [16, 18] (Fig. 3). Given a set of real and detected homology groups, for each true homology group, THG, we find the detected homology group, DHG, which has the largest overlap with the THG. Then we consider true positives (*tp*) as the number of proteins in both THG and DHG, false negatives (*fn*) as the number of proteins in THG but not in DHG, and false positives (*fp*) as the number of proteins avilable in DHG but not in THG. Then TP, FP and FN are defined as the summation of the *tp’*s, *fp’*s and *fn’*s over all true homology groups, respectively. Finally, the recall, precision and F-score measures are calculated as follows:

$$ \mathrm{recall}=\mathrm{TP}/\left(\mathrm{TP}+\mathrm{FN}\right) $$

$$ \mathrm{precision}=\mathrm{TP}/\left(\mathrm{TP}+\mathrm{FP}\right) $$

$$ \mathrm{F}\hbox{-} \mathrm{score}=2\times \left(\mathrm{Recall}\times \mathrm{Precision}\right)/\left(\mathrm{Recall}+\mathrm{Precision}\right) $$

Recall represents the ability of the method to put the true homologs together in one group, precision shows its ability to separate the non-homologs, and the F-score is the harmonic mean of these two measures combining them in one. There is always a trade-off between recall and precision, since detecting more TPs often leads to some FPs.

In the following experiments, we need to know the real groups in various datasets to serve as a ground truth for evaluation. For the *S.cerevisiae* datasets and the single *E.coli* dataset, the real groups are defined based on the locus tags of the proteins extracted from the GenBank files (Additional file 2). For *A.thaliana* datasets the real groups are defined based on the gene identifiers which end with .1, for example AT3G54340.1, which correspond to the first annotated isoform of the genes. For the single Metazoa dataset, we used the identifiers of the 70 protein families of OrthoBench as the real group identifiers.

## Results and discussion

Here, we present results demonstrating the accuracy, scalability, efficiency and applicability of PanTools for detecting homology in large proteomes of bacteria, fungi, plants and Metazoa (Additional file 1: Table S1). We compare PanTools to the BLAST-based orthology detector OrthoFinder [16] and to DIAMOND-based PanX [19], a pipeline dedicated to microbial data (Additional file 1: Tables S2–S5). First we evaluated the methods on OrthoBench [18], a public benchmark of curated protein families from 12 metazoans. Unfortunately, we were not able to run PanX on this data (M12), as this benchmark only provides the protein sequences but not the gene sequences PanX requires. Next, we tested scalability on 5 datasets of increasing size compiled from 93 *Saccharomyces cerevisiae* strains [20] and 5 datasets compiled from 19 *Arabidopsis thaliana* accessions [21]. Additionally, we compared the performance of PanTools and PanX on a large dataset of 600 *Escherichia coli*; we did not run OrthoFinder, as we estimated it would need ~ 5000 h on this dataset. Finally, we studied the effect of evolutionary distance on homology detection using 12 Brassicaceae species proteomes. Experiments were executed on an Ubuntu 14.04 server, Intel® Xeon® X5660@2.8GHz, with 64GB RAM using 16 processing cores and 32GB of RAM disk.

### PanTools is adaptable to handle varying degrees of input divergence

**Fig. 4:**
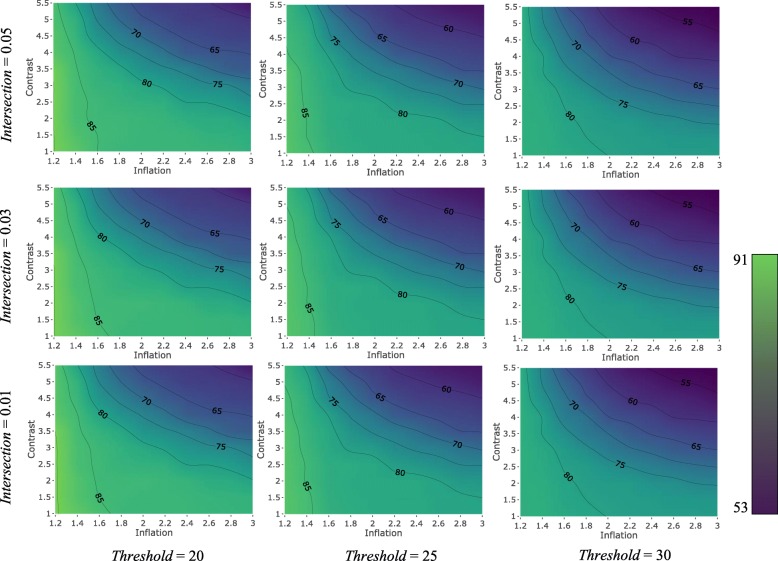
The effect of intersection rate, similarity threshold, contrast and inflation rate, on the recall of PanTools. Each contour plot belongs to a pair of intersection and threshold values, with the x and y axis representing inflation and contrast parameters

**Fig. 5:**
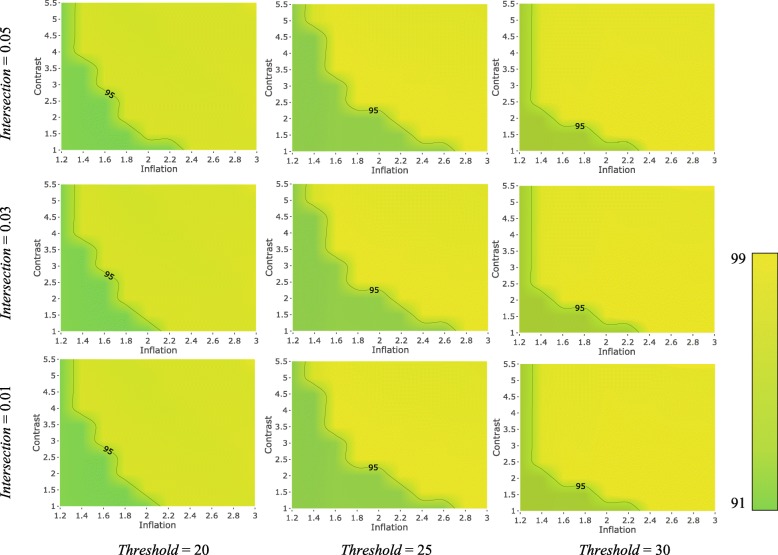
The effect of intersection rate, similarity threshold, contrast and inflation rate, on the precision of PanTools. Each contour plot belongs to a pair of intersection and threshold values, with the x and y axis representing inflation and contrast parameters

PanTools has four main parameters that affect the homology clustering: intersection rate, similarity threshold, contrast and MCL inflation. To examine the general effect of these parameters on the accuracy of the method on proteomes of diverged species, we used the set of 1695 proteins from the OrthoBench. Figures 4 and 5 present contour plots illustrating the effect of these four parameters on the recall and precision of PanTools, respectively; lighter colors represent higher values.

The first parameter, intersection rate (*I*) (in the range of 0.01–0.1), determines the minimum number of hexamers that two proteins need to have in common to be considered intersecting proteins in order to be selected for exact alignment. This number is calculated as the product of the intersection rate and the total number of hexamers of the shorter protein. In general, by choosing lower intersection values the number of pairwise alignments and, in turn, the resource consumption of the program increases, significantly. The lower the intersection value, the higher the recall and the lower the precision.

The second parameter affecting the clustering is the similarity threshold (*T*) (in the range of 25–99). Two proteins are considered similar if the normalized similarity score of their local alignment exceeds this threshold. Lower thresholds increase the number of detected similarities, boosting the sensitivity of the homology detection. So, the lower the threshold, the higher the recall, but the lower the precision.

The connectivity components of the similarity graph (similarity components) are the candidate homology groups which are then passed to the MCL clustering algorithm to be possibly split into more specific homology groups. To increase the granularity of the clustering and split the similarity components into a larger number of groups, we choose greater MCL inflations (*M*). Finally, we raise the scores to the power of the contrast parameter (*C*) to increase the contrast between the final similarity scores. Like for *I* and *T*, the lower the inflation and/or contrast, the higher the recall and the lower the precision.

The resulting F-scores (Additional file 1: Figure S1) suggest that higher values of the four parameters are not desirable for grouping the proteome of these distant species. In support of this, we observed that increasing the parameter values improves the F-score of the method when analyzing the proteomes of closely related species.

Based on these observations, we experimentally optimized 8 groups of default parameter settings (*d1*-*d8*), ranging from strict to relaxed by linearly decreasing the 4 mentioned parameters (Additional file 1: Table S6). This allows the user to fine-tune the settings for different types of datasets and/or downstream applications. We recommend users to either use Table S6 to choose appropriate settings based on the divergence of the proteomes or try multiple settings and pick one based on the desired resolution from one-to-one orthologs to multi-gene families. In our experiments, we used the most strict setting (*d1*) for the closely related strains of *E.coli* and *S.cerevisiae*, the next strict setting (*d2*) for *A.thaliana* datasets, and the most relaxed setting (*d8*) for the OrthoBench data.

### PanTools is efficient and accurate on OrthoBench data

OrthoBench is a resource of 70 curated eukaryotic protein families from 12 metazoans which was established to assess the performance of TreeFam [22], eggNOG [23], OrthoDB [24], OrthoMCL [25], and OMA [26]. We call this benchmark M12 in the rest of this paper. The homology relationships between these protein families are difficult to detect due to differences in their rate of evolution, domain architecture, low-complexity regions/repeats, lineage-specific losses/duplications, and alignment quality [18].

We compared the performance of PanTools to that of OrthoFinder, which previously showed the highest accuracy on this benchmark data. We first created a mapping from the 1695 OrthoBench proteins to the 404,657 proteins of the 12 metazoans available in Ensembl release 60. We then ran PanTools and OrthoFinder independently on these 12 complete proteomes and calculated the recall, precision and F-score using the same procedure as proposed for OrthoFinder. In this experiment, PanTools achieved the same recall as OrthoFinder but at a remarkably higher precision, resulting in a 3% higher overall F-score of 85.5%. Additionally, there were significant differences in run-times. Running on 16 cores, PanTools terminated after 2 h and OrthoFinder after 77.6 h.

### PanTools scales to large eukaryotic datasets and maintains accuracy

**Fig. 6:**
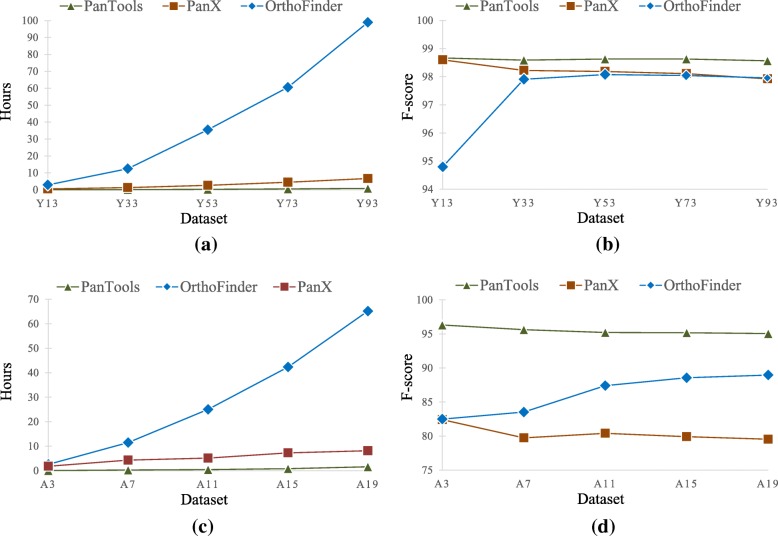
**a** The run-time and **b** the F-score of the three methods on the 5 *S. cerevisiae* datasets. **c** The run-time and **d** the F-score of the three methods on the 5 *A. thaliana* datasets

To examine the scalability of our method to large eukaryotic datasets, we first ran it on 5 datasets of *Saccharomyces cerevisiae* (Y3, Y13, …, Y93) and on 5 datasets of *Arabidopsis thaliana* accessions (A3, A7, …, A19) with an increasing number of proteomes. We compared the run-time and accuracy (F-score) of PanTools to those of OrthoFinder and PanX (Fig. 6).

On the largest yeast dataset (Y93), PanTools was 112 times faster than OrthoFinder (0.9 h vs. 4 days) and 7.6 times faster than PanX, with a slightly higher F-score. Similarly, on the largest Arabidopsis dataset (19 accessions), PanTools was 42 times faster (1 h vs. 2.7 days) than OrthoFinder and 5.2 times faster than PanX while maintaining its higher F-score. Overall, OrthoFinder starts with a low accuracy but seems to level out at a higher value as the number of proteomes grows, albeit at the cost of drastic increase in run-time. Although PanX was almost as accurate as OrthoFinder on the *S.cerevisiae* data, its accuracy fell below that of OrthoFinder on the *A. thaliana* data, likely because plants have more diverse proteomes than the bacteria PanX was designed for.

### PanTools is applicable to large microbial datasets

To compare the performance of our approach to PanX, a recently published tool dedicated to the microbial data, we applied both tools to the proteomes of 600 *E.coli* strains downloaded from GenBank (Additional file 2). Both PanX and PanTools processed this large dataset in ~ 15 h, resulting in F-scores of 71.6 and 72.9, respectively. In this experiment, we ran PanX in divide-and-conquer mode to speed it up.

### PanTools significantly reduces the number of pairwise comparisons

The efficiency of PanTools is due to its *k*-mer-based approach, which significantly reduces the number of fruitless protein alignments. Table 1 shows that the numbers of pairwise comparisons in different experiments are thousands-fold less than what is needed in a naïve all-pairs approach.

**Table 1:** The number of PanTools comparisons compared to a naïve all-pairs approach

Dataset	Naïve (millions)	PanTools (thousands)	Fold decrease
Y13	2472	507	4874
Y33	15,979	3415	4679
Y53	41,181	8888	4633
Y73	78,121	16,937	4613
Y93	126,519	27,494	4602
A3	4284	508	8435
A7	23,225	2889	8038
A11	57,382	7229	7938
A15	105,111	12,904	8146
A19	169,570	21,022	8066
M12	81,873	20,094	4074
E600	4,993,364	926,638	5389

To scale to hundreds of eukaryotic or thousands of prokaryotic proteomes using reasonable amount of resources, there were two main limitations to be resolved: first, the local sequence alignment of proteins, which we tried to mitigate by distributing the intersecting pairs among multiple threads to be aligned in parallel; second, the size of the data structure used for detecting the intersecting proteins, which grows linearly with the size of the input data. To reduce the memory needs, currently we ignore extremely abundant and rare hexamers, which are less informative. By using space-efficient data structures, for example MinHash sketches [27], we may be able to further decrease the memory consumption of the program.

### PanTools reproduces the majority of groups detected by other tools

In all experiments, PanTools was able to perfectly reproduce the majority of the groups detected by OrthoFinder and PanX. Table 2 shows the percentage of the groups generated by OrthoFinder and PanX which have an identical counterpart in the PanTools groups. Generally, the overlap decreases as the size of data grows, because the probability of having exactly identical groups drops, although the corresponding groups have highly similar compositions.

**Table 2:** The percentage of OrthoFinder and PanX groups that PanTools reproduce

Dataset	Reproduced	Reproduced
	OrthoFinder groups	PanX groups
Y13	94.9%	96.3%
Y33	94.9%	95.5%
Y53	93.9%	94.8%
Y73	93.6%	94.5%
Y93	93.3%	93.8%
A3	72.1%	80.1%
A7	64.9%	71.5%
A11	64.9%	69.1%
A15	64.8%	72.5%
A19	64.6%	79.8%
M12	76.3%	–
E600	–	59.7%

### Parameters can affect the performance of different application scenarios

To investigate the effect of the 8 suggested parameter sets (from strict to relaxed) on homology clustering, we used a large proteome of 12 phylogenetically diverse Brassicaceae species, including the model plant *Arabidopsis thaliana*, plus *Vitis vinifera* as an outgroup. We specifically considered four genes with different copy numbers in *A.thaliana*, including three MADS-box genes – the floral homeotic protein APETALA 3 (AP3), the floral homeotic protein AGAMOUS (AG) and the flowering locus C (FLC) – and one housekeeping gene: the ubiquitin extension protein 1 (UBQ1), and looked into the composition of their homology groups detected by PanTools using the 8 parameter settings from strictest (*d*1) to the most relaxed (*d*8). Each column of Table 3 represents a homology group and each entry reflects the count of homologs of the genes AP3, AG, FLC and UBQ1 from different species in that group.

**Table 3:** Counts of the homologs of 4 genes from Brassicaceae species in each homology group

	AP3								AG								FLC								UBQ1							
	(AT3G54340)								(AT4G18960)								(AT5G10140)								(AT3G52590)							
	*d1*	*d2*	*d3*	*d4*	*d5*	*d6*	*d7*	*d8*	*d1*	*d2*	*d3*	*d4*	*d5*	*d6*	*d7*	*d8*	*d1*	*d2*	*d3*	*d4*	*d5*	*d6*	*d7*	*d8*	*d1*	*d2*	*d3*	*d4*	*d5*	*d6*	*d7*	*d8*
*A. thaliana*	1	1	1	1	1	1	1	1	1	1	1	1	3	4	8	27	1	1	1	2	4	6	8	36	2	2	2	2	15	16	17	17
*A. lyrata*	1	1	1	1	1	1	1	3	1	1	1	1	4	4	7	24	0	1	1	1	4	5	5	32	0	0	0	0	19	20	21	22
*C. rubella*	1	1	1	1	1	1	1	2	1	1	1	1	3	4	8	24	0	1	1	1	2	3	4	33	2	2	2	2	15	15	18	19
*M. maritima*	1	1	1	1	1	1	1	3	1	1	1	1	4	4	7	24	0	1	1	1	4	4	6	34	2	2	2	2	12	12	16	18
*D. sophiades*	1	1	1	1	1	1	1	3	1	1	1	1	4	4	9	26	0	1	1	1	7	9	12	42	2	2	2	2	11	12	12	12
*S. irio*	0	1	1	1	1	1	3	6	0	0	2	3	8	8	10	26	0	0	0	2	4	4	11	36	3	3	3	3	13	14	17	20
*M. perfoliatum*	1	1	1	1	1	1	1	2	1	1	1	1	4	4	8	28	0	1	1	1	3	5	8	35	3	3	3	3	13	13	18	21
*T. salsuginea*	1	1	1	1	1	1	1	3	1	1	1	1	4	4	8	27	0	1	1	1	5	5	7	33	2	2	2	2	12	13	13	13
*T. halophila*	0	0	2	2	2	2	2	3	0	0	0	4	6	9	13	41	0	0	0	0	0	4	4	48	4	4	4	4	15	15	15	15
*A. alpina*	1	1	1	1	1	1	2	3	0	1	2	2	5	7	9	21	0	0	1	3	6	7	11	30	5	6	6	7	17	17	21	23
*E. syriacum*	1	1	1	1	1	1	1	2	0	0	0	0	2	3	7	21	0	0	1	1	3	5	9	31	3	3	3	3	18	20	21	22
*A. arabicum*	0	1	1	1	2	3	4	5	0	1	2	2	8	11	13	24	0	1	2	3	4	5	7	25	3	3	3	3	12	12	12	13
*V. vinifera*	0	0	0	0	1	1	1	4	0	0	1	1	4	4	8	26	0	0	0	0	0	0	3	34	2	2	3	3	7	9	9	10

Each column represents a detected homology group with one of the default settings (*d*1-*d*8)

With all settings, we detected a single *AP3* homolog in Arabidopsis, which indicates that this MADS-box gene is significantly differentiated from other MADS-box genes. We also found unique orthologues for most of the other species.

We detected a single ortholog of AG until *d*5, after which we also identify its ancient paralogs Shatterproof 1 and 2 (SHP1/2). The duplication that gave rise to the split between AG and SHP1/2 is quite old (the gamma triplication shared by most eudicot species). At *d*6 we also detect STK which comes from an even earlier duplication (perhaps at the origin of angiosperms) [28]. At *d*7 and *d*8 we identify many of the various MADS-box genes across different lineages.

FLC is alone until *d*4, where the transposition duplicate MAF1 (but not yet members of the MAF2–5 clade) is added. Then MAF2–5 members derived from the At-alpha WGD (whole genome duplication) from FLC come up, followed by inclusion of the tandem expansion of these genes. At subsequent settings, we start picking up other MADS-box genes.

UBQ1 is a house-keeping gene that was duplicated by the ancient whole genome duplication (WGD) At-alpha shared across the Brassicaceae (PGDD database) [29]. Our method recovered both the ortholog and its in-paralog (UBQ2) even using the strictest setting (*d*1), meaning that these genes are very similar despite having diverged around 40 mya. Thus, the function of the two genes is likely highly conserved. From *d*5 on, PanTools identifies other, more distantly related homologs and ultimately (*d*8) all members of the larger family (UBQ1-UBQ14) plus a few related genes.

Table 4 shows the distribution of the normalized similarity scores in each of the detected homology groups. It is clear that more relaxed settings allow including more diverse pairs of homologs, which are less similar in the final clusters.

**Table 4:** Minimum, maximum and average of normalized similarity scores in the homology group of 4 genes using 8 different settings

Gene name (ID)		*d1*	*d2*	*d3*	*d4*	*d5*	*d6*	*d7*	*d8*
AP3 (AT3G54340)	Min	95.1	88.1	79.3	79.3	57.5	57.5	57.5	25.0
	Avg	97.0	95.5	91.7	91.7	87.2	87.2	87.2	38.7
	Max	98.8	98.8	98.8	98.8	98.8	98.8	98.8	100.0
AG (AT4G18960)	Min	95.1	85.2	75.7	75.7	56.2	45.0	35.0	25.0
	Avg	96.7	93.6	90.1	89.3	82.6	62.7	52.2	38.7
	Max	99.5	99.5	99.5	98.0	99.5	100.0	100.0	100.0
FLC (AT5G10140)	Min	–	85.1	79.9	65.0	55.0	45.0	35.0	25.0
	Avg	–	87.3	85.2	75.1	68.3	62.7	52.2	38.7
	Max	–	94.4	94.4	100.0	100.0	100.0	100.0	100.0
UBQ1 (AT3G52590)	Min	98.2	96.9	81.7	81.7	57.2	45.0	35.0	25.1
	Avg	99.6	99.3	99.6	99.6	97.2	78.3	72.0	68.5
	Max	100.0	100.0	100.0	100.0	100.0	100.0	100.0	100.0

## Conclusion

We presented an efficient method for detecting homology groups across a large number of individuals and/or species. To make homology detection efficient we adopted a *k*-mer-based approach, which substantially reduces the number of pairwise comparisons. Specifically, we first count the number of peptide hexamers two proteins share, and only if this number is high enough, we perform a local alignment of the so-called intersecting proteins to calculate their exact similarity score.

We clearly observed a trade-off between recall and precision of the homology inference. Favoring recall or precision strongly depends on the application [30]. In a phylogenetic study one may specifically be interested in identifying precise one-to-one orthologs, while others may want to capture a complete protein family to achieve insights into gene-duplication events across species. The four defined parameters (and the 8 default settings) give users the flexibility to control the program’s behavior. It is important to note that different types of genes may be under different selection pressures and constraints and have different evolutionary dynamics. Thus, the optimal parameter setting will depend both on the specific gene and on the desired application, as demonstrated by the four genes across the Brassicaceae.

As we store the homology groups in the pan-genome, it is possible to query the pan-genome graph database for statistics on, for example, the size of the homology groups, the copy number of the genes and the conservation rate of the proteins in different groups. In the future, we will extend PanTools with additional functionality to exploit this pan-genome database for comparative genomics on large collections of complex genomes.

## Additional files

Additional file 1: Supplementary methods, tables, and figures Caption. (DOCX 1107 kb)

Additional file 2: Data description. (XLSX 52 kb)
