# Supplementary material for: Efficient inference of homologs in large eukaryotic pan-proteomes
Source: BMC Bioinformatics. 2018 Sep 26;19:340. doi: 10.1186/s12859-018-2362-4 (PMC6158922; doi:10.1186/s12859-018-2362-4)
Supplement: Supplementary file 1 — Supplementary methods, tables, and figures Caption. (DOCX 1107 kb) [file 12859_2018_2362_MOESM1_ESM.docx]

Supplementary materials

**Efficient Inference of Homologs in Large Eukaryotic Pan-proteomes**

**Corresponding author:** Siavash Sheikhizadeh Anari, Bioinformatics Group, Wageningen University, The Netherlands

siavash.sheikhizadehanari@wur.nl

Dick de Ridder, Bioinformatics Group, Wageningen University, The Netherlands

dick.deridder@wur.nl

M. Eric Schranz, Biosystematics Group, Wageningen University, The Netherlands

eric.schranz@wur.nl

Sandra Smit, Bioinformatics Group, Wageningen University, The Netherlands

sandra.smit@wur.nl

**Construction of the pan-genome**

Here we present instructions for running the homology detection functionality of PanTools. First, the PanTools package should be cloned to the home directory from GitHub:

cd

git clone https://github.com/sheikhizadeh/pantools

You should already have installed Java (JDK 1.8 or higher) and the latest version of MCL clustering program on your machine and have added the path to their executables to the PATH shell environment variable.

**Constructing the proteome layer of the pan-genome**

To construct the proteome layer of a pan-genome from a set of protein sequences, collect the paths to all the protein files in FASTA format in a text file (for example, proteins.txt) which contains these lines:

$HOME/proteins/p1.faa

$HOME/proteins/p2.faa

$HOME/proteins/p3.faa

Then run:

java -jar pantools/dist/pantools.jar build_panproteome -dp ./DB -pf ./proteins.txt

To start homology detection using 4 threads and the most relaxed setting (8) type:

java -jar pantools/dist/pantools.jar group -dp ./DB -tn 4 -rn 8

Alternatively, you can start from genomes and GFF files and build a complete pan-genome. First, you need to construct the genome layer of a pan-genome by collecting the path to all the genome files in FASTA format in a text file (for example, genomes.txt) which contains these lines:

$HOME/genomes/g1.fna

$HOME/genomes/g2.fna

$HOME/genomes/g2.fna

Then:

java -jar pantools/dist/pantools.jar build_pangenome -dp ./DB -gf ./genomes.txt

The genomes’ annotation should be added to build the annotation layer of the pan-genome by giving the path to the GFF file for each genome as indicated by the number at the start of each line (for example, in annotations.txt) which contains these lines:

1 $HOME/annotations/a1.gff

2 $HOME/annotations/a2.gff

3 $HOME/annotations/a3.gff

Then run:

java -jar pantools/dist/pantools.jar add_annotations -dp ./DB -af ./annotations.txt

Now, the proteins are annotated in the pan-genome and homology detection can be started by:

java -jar pantools/dist/pantools.jar group -dp ./DB -tn 4 -rn 8

**Choice of K**

Here, we calculate the smallest *k* value which keeps the probability of random occurrences of a *k*-mer below a desirable probability *p*. Given sequences *S* of length *L* from an alphabet of size *α*, the probability of a given *k*-mer from this alphabet being present at a given position of *S* by chance is:

(1) $x=\frac{1}{\alpha^{k}}$

then, the probability of the given *k*-mer being absent at a given position of *S* is:

(2) $1-x$

and the probability that the *k*-mer occurs nowhere in *S* will be:

(3) $\left( 1-x \right)^{n}$, where *n* is the number of *k*-mers in sequence S: *L*$-k+1$

so, the probability that it occurs somewhere in *S* would be:

(4) ${1-\left( 1-x \right)}^{n}$

Using the Taylor (or Maclaurin) series when $\left| x \right|\ll1$, which holds here as $\alpha^{k}\gg1$:

$$\ln\left( 1-x \right)=-x-\frac{x^{2}}{2}-\frac{x^{3}}{3}-\frac{x^{4}}{4}-\ldots\approx-x$$

$$n\ln\left( 1-x \right)\approx-nx$$

$$\left( 1-x \right)^{n}\approx exp(-nx)$$

So, the probability (4) is well approximated by:

(5) $1-exp\left( -xn \right)$

setting this probability less than the desired value *p* we will have:

$$1-exp\left( -xn \right) <p$$

$-xn>ln(1-p)$

$$x<\frac{ln(1-p)}{-n}$$

substituting *x* gives:

$$\frac{1}{\alpha^{k}}<\frac{ln(1-p)}{-n}$$

(6) $k \geq\left\lceil\log_{\alpha} \frac{-n}{\ln\left( 1-p \right)} \right\rceil$

For peptide sequences *α =* 20*,* and considering $n\approx L = 30,000$ the length of the largest known protein, and setting *p =* 0.001, the smallest suitable *k* is 6.

**Experiments**

We demonstrate the accuracy, scalability, efficiency and applicability of PanTools on 12 datasets of bacteria, fungi, plants and Metazoa. Y13-Y93 are 5 datasets of increasing size compiled from 93 *Saccharomyces cerevisiae* strains and A3-A19 are 5 datasets compiled from 19 *Arabidopsis thaliana* accessions. M12 is the OrthoBench data from 12 metazoans and E600 a large dataset of 600 *Escherichia coli* strains.

For most of these data sets we selected a subset of the total proteins for evaluation of the methods (Table S1), because we were not able to establish a ground truth for all. Columns “Intersecting” and “Similar” give the number of intersecting resp. similar pairs of proteins in the constructed pan-genome.

Table S1. Some statistics about the pan-genomes constructed by PanTools for homology detection in the 12 mentioned datasets.

| **Dataset** | ***k*-mers** | **All**  **proteins** | **Selected**  **proteins** | **Intersecting** | **Similar** |
| --- | --- | --- | --- | --- | --- |
| **Y13** | 2,634,965 | 70,312 | 61,414 | 507,164 | 423,162 |
| **Y33** | 2,722,436 | 178,769 | 156,135 | 3,414,746 | 2,872,902 |
| **Y53** | 2,794,291 | 286,987 | 250,649 | 8,887,967 | 7,486,596 |
| **Y73** | 2,947,823 | 395,274 | 345,333 | 16,936,559 | 14,275,904 |
| **Y93** | 3,027,750 | 503,028 | 439,573 | 27,494,049 | 23,189,200 |
| **A3** | 8,324,590 | 92,564 | 92,564 | 507,874 | 139,384 |
| **A7** | 8,861,259 | 215,523 | 215,523 | 2,889,361 | 888,305 |
| **A11** | 9,167,464 | 338,769 | 338,769 | 7,228,520 | 2,273,496 |
| **A15** | 9,425,939 | 458,499 | 458,499 | 12,903,576 | 4,197,580 |
| **A19** | 9,621,923 | 582,357 | 582,357 | 21,021,793 | 6,853,404 |
| **M12** | 29,193,967 | 404,657 | 404,657 | 20,094,137 | 12,942,693 |
| **E600** | 7,580,644 | 3,160,178 | 688,160 | 926,638,469 | 734,423,473 |

Table S2 shows the number of groups detected by each of the three tools, the number of real groups and the running time of tools in hours. For the Y datasets and the E600 dataset, the real groups are determined by the valid locus tags of the proteins extracted from the GenBank files. For A datasets, the real groups are the gene identifiers which end with .1, corresponding to the first annotated isoform of the genes. For M12 we used the identifier of the known 70 protein families in the OrthoBench as the real group identifiers.

Table S2. Results of running PanTools, OrthoFinder and PanX on the 12 datasets. Run-times are presented in hours.

| **Dataset** | **PanTools**  **groups** | **OrthoFinder**  **groups** | **PanX**  **groups** | **Real**  **groups** | **PanTools**  **run-time** | **OrthoFinder**  **run-time** | **PanX**  **run-time** |
| --- | --- | --- | --- | --- | --- | --- | --- |
| **Y13** | 4,990 | 4,746 | 4,830 | 4,894 | 0.02 | 2.86 | 0.52 |
| **Y33** | 5,078 | 4,853 | 4,849 | 4,906 | 0.11 | 12.50 | 1.28 |
| **Y53** | 5,151 | 4,886 | 4,855 | 4,909 | 0.28 | 35.53 | 2.65 |
| **Y73** | 5,199 | 4,889 | 4,857 | 4,910 | 0.51 | 60.60 | 4.47 |
| **Y93** | 5,257 | 4,886 | 4,867 | 4,911 | 0.88 | 98.95 | 6.72 |
| **A3** | 32,236 | 29,231 | 30,065 | 30,971 | 0.05 | 2.58 | 1.77 |
| **A7** | 35,083 | 31,368 | 31,356 | 31,037 | 0.25 | 11.47 | 4.28 |
| **A11** | 36,842 | 33,367 | 32,048 | 31,046 | 0.43 | 25.02 | 5.12 |
| **A15** | 38,292 | 34,192 | 32,637 | 31,047 | 0.79 | 42.30 | 7.28 |
| **A19** | 39,579 | 34,896 | 33,095 | 31,049 | 1.55 | 65.20 | 8.13 |
| **M12** | 171 | 147 | - | 70 | 1.96 | 77.60 | - |
| **E600** | 10,196 | - | 7,843 | 10,152 | 15.65 | - | 15.48 |

Table S3. Accuracy of PanTools on the 12 datasets.

| **Pan-genome** | **TP** | **FP** | **FN** | **Recall** | **Precision** | **F_score** |
| --- | --- | --- | --- | --- | --- | --- |
| **Y13** | 61,101 | 1,339 | 313 | 99.5 | 97.9 | 98.7 |
| **Y33** | 155,361 | 3,659 | 774 | 99.5 | 97.7 | 98.6 |
| **Y53** | 249,402 | 5,679 | 1,247 | 99.5 | 97.8 | 98.6 |
| **Y73** | 343,636 | 7,852 | 1,697 | 99.5 | 97.8 | 98.6 |
| **Y93** | 437,305 | 10,435 | 2,268 | 99.5 | 97.7 | 98.6 |
| **A3** | 90,033 | 4,353 | 2,531 | 97.3 | 95.4 | 96.3 |
| **A7** | 207,675 | 11,168 | 7,848 | 96.4 | 94.9 | 95.6 |
| **A11** | 325,625 | 19,646 | 13,144 | 96.1 | 94.3 | 95.2 |
| **A15** | 440,596 | 26,837 | 17,903 | 96.1 | 94.3 | 95.2 |
| **A19** | 558,677 | 34,412 | 23,680 | 95.9 | 94.2 | 95.1 |
| **M12** | 1,328 | 135 | 315 | 80.8 | 90.8 | 85.5 |
| **E600** | 665,167 | 472,215 | 22,993 | 96.7 | 58.5 | 72.9 |

Table S4. Accuracy of OrthoFinder on the 11 datasets.

| **Pan-genome** | **TP** | **FP** | **FN** | **Recall** | **Precision** | **F_score** |
| --- | --- | --- | --- | --- | --- | --- |
| **Y13** | 61,356 | 6,678 | 58 | 99.9 | 90.2 | 94.8 |
| **Y33** | 155,945 | 6,457 | 190 | 99.9 | 96.0 | 97.9 |
| **Y53** | 250,363 | 9,532 | 286 | 99.9 | 96.3 | 98.1 |
| **Y73** | 344,989 | 13,859 | 344 | 99.9 | 96.1 | 98.0 |
| **Y93** | 439,192 | 17,943 | 381 | 99.9 | 96.1 | 98.0 |
| **A3** | 86,363 | 34,435 | 2,246 | 97.5 | 71.5 | 82.5 |
| **A7** | 205,912 | 74,595 | 6,657 | 96.9 | 73.4 | 83.5 |
| **A11** | 325,099 | 82,904 | 10,849 | 96.8 | 79.7 | 87.4 |
| **A15** | 444,098 | 98,963 | 15,102 | 96.7 | 81.8 | 88.6 |
| **A19** | 560,002 | 119,627 | 19,534 | 96.6 | 82.4 | 88.9 |
| **M12** | 1,328 | 243 | 315 | 80.8 | 84.5 | 82.6 |

Table S5. Accuracy of PanX on the 11 datasets.

| **Pan-genome** | **TP** | **FP** | **FN** | **Recall** | **Precision** | **F_score** |
| --- | --- | --- | --- | --- | --- | --- |
| **Y13** | 61,302 | 1,717 | 19 | 100.0 | 97.3 | 98.6 |
| **Y33** | 155,800 | 5,566 | 58 | 100.0 | 96.6 | 98.2 |
| **Y53** | 250,144 | 9,176 | 68 | 100.0 | 96.5 | 98.2 |
| **Y73** | 344,660 | 13,193 | 79 | 100.0 | 96.3 | 98.1 |
| **Y93** | 438,691 | 18,460 | 108 | 100.0 | 96.0 | 97.9 |
| **A3** | 87,047 | 35,756 | 1,387 | 98.4 | 70.9 | 82.4 |
| **A7** | 208,010 | 101,599 | 4,072 | 98.1 | 67.2 | 79.7 |
| **A11** | 328,418 | 153,371 | 6,688 | 98.0 | 68.2 | 80.4 |
| **A15** | 448,690 | 216,306 | 9,317 | 98.0 | 67.5 | 79.9 |
| **A19** | 568,800 | 280,513 | 11,987 | 97.9 | 67.0 | 79.5 |
| **E600** | 667,680 | 513,833 | 15,785 | 97.7 | 56.5 | 71.6 |

**Pre-cooked default parameters**

Table S6. The values of each of the four parameters *I*, *T*, *M* and *C* in each of the default pre-cooked set of parameters.

| **Parameter** | ***d1*** | ***d2*** | ***d3*** | ***d4*** | ***d5*** | ***d6*** | ***d7*** | ***d8*** |
| --- | --- | --- | --- | --- | --- | --- | --- | --- |
| Similarity threshold (T*)* | 95 | 85 | 75 | 65 | 55 | 45 | 35 | 25 |
| Intersection rate (*I*) | 0.09 | 0.08 | 0.07 | 0.06 | 0.05 | 0.04 | 0.03 | 0.02 |
| MCL inflation (*M*) | 9.6 | 8.4 | 7.2 | 6.0 | 4.8 | 3.6 | 2.4 | 1.2 |
| Contrast (*C*) | 8 | 7 | 6 | 5 | 4 | 3 | 2 | 1 |

**F-score counter plot**

Figure S1. The effect of intersection rate, similarity threshold, contrast and inflation rate, on the **F-score** of PanTools. Each contour plot belongs to a pair of intersection and threshold values, with the x and y axis representing inflation and contrast parameters.
